# Blinded Validation of Breath Biomarkers of Lung Cancer, a Potential Ancillary to Chest CT Screening

**DOI:** 10.1371/journal.pone.0142484

**Published:** 2015-12-23

**Authors:** Michael Phillips, Thomas L. Bauer, Renee N. Cataneo, Cassie Lebauer, Mayur Mundada, Harvey I. Pass, Naren Ramakrishna, William N. Rom, Eric Vallières

**Affiliations:** 1 Breath Research Laboratory, Menssana Research Inc, 211 Warren St, Newark, NJ, 07103, United States of America; 2 Department of Medicine, New York Medical College, Valhalla, NY, United States of America; 3 Christiana Care Health System, Newark, DE, United States of America; 4 Schmitt & Associates, 211 Warren Street, Newark, NJ, 07103, United States of America; 5 New York University Langone Medical Center, New York, NY, United States of America; 6 University of Florida Health Cancer Center at Orlando Health, Orlando, FL, United States of America; 7 Swedish Cancer Institute, 1101 Madison Suite 900, Seattle, WA, 98104, United States of America; University of Athens Medical School, SWITZERLAND

## Abstract

**Background:**

Breath volatile organic compounds (VOCs) have been reported as biomarkers of lung cancer, but it is not known if biomarkers identified in one group can identify disease in a separate independent cohort. Also, it is not known if combining breath biomarkers with chest CT has the potential to improve the sensitivity and specificity of lung cancer screening.

**Methods:**

*Model-building phase (unblinded)*: Breath VOCs were analyzed with gas chromatography mass spectrometry in 82 asymptomatic smokers having screening chest CT, 84 symptomatic high-risk subjects with a tissue diagnosis, 100 without a tissue diagnosis, and 35 healthy subjects. Multiple Monte Carlo simulations identified breath VOC mass ions with greater than random diagnostic accuracy for lung cancer, and these were combined in a multivariate predictive algorithm. *Model-testing phase (blinded validation)*: We analyzed breath VOCs in an independent cohort of similar subjects (n = 70, 51, 75 and 19 respectively). The algorithm predicted discriminant function (DF) values in blinded replicate breath VOC samples analyzed independently at two laboratories (A and B). *Outcome modeling*: We modeled the expected effects of combining breath biomarkers with chest CT on the sensitivity and specificity of lung cancer screening.

**Results:**

*Unblinded model-building phase*. The algorithm identified lung cancer with sensitivity 74.0%, specificity 70.7% and C-statistic 0.78. *Blinded model-testing phase*: The algorithm identified lung cancer at Laboratory A with sensitivity 68.0%, specificity 68.4%, C-statistic 0.71; and at Laboratory B with sensitivity 70.1%, specificity 68.0%, C-statistic 0.70, with linear correlation between replicates (r = 0.88). In a projected outcome model, breath biomarkers increased the sensitivity, specificity, and positive and negative predictive values of chest CT for lung cancer when the tests were combined in series or parallel.

**Conclusions:**

Breath VOC mass ion biomarkers identified lung cancer in a separate independent cohort, in a blinded replicated study. Combining breath biomarkers with chest CT could potentially improve the sensitivity and specificity of lung cancer screening.

**Trial Registration:**

ClinicalTrials.gov NCT00639067

## Introduction

The modern era of breath testing dawned in 1971, when Linus Pauling first reported that normal human breath contains large numbers of volatile organic compounds (VOCs) in low concentrations [1]. Subsequent researchers have attempted to employ breath VOCs as disease biomarkers with varying degrees of success. The U.S. Food & Drug Administration (FDA) has approved a small number of breath tests for clinical use (e.g. breath nitric oxide for airways inflammation [2]), but FDA has not yet approved a breath test for lung cancer. Despite 30 years of research resulting in more than 300 relevant publications, no single breath VOC has emerged as a clinically useful biomarker of lung cancer when employed alone. However, several breath VOCs appear to provide moderately accurate biomarkers that could potentially identify lung cancer if combined with one another in a multifactorial algorithm [3].

In seeking breath biomarkers of lung cancer, researchers have employed a wide range of different tools including VOC separation methods [e.g. gas chromatography mass spectrometry (GC MS) [3–6], non-separative detectors (e.g. electronic noses and chemosensors [7–9]), analysis of expired breath condensate [10], measurement of breath temperature [11], and sniffer dogs [12]. Analysis of breath VOCs with analytical instruments employing 2-dimensional GC has revealed a complex matrix of ~2,000 different VOCs in a single sample [13,14]. The resulting flood of information has necessitated use of data management tools for metabolomic analysis that were originally developed for genomics and proteomics. This has been accompanied by an increased risk of false discovery of biomarkers that can arise when a multivariate model over-fits large number of candidate breath VOCs to a small number of test subjects, a pitfall that has been termed “voodoo correlations”, or “seeing faces in the clouds” [15].

Despite these concerns, breath biomarkers of lung cancer have been proposed as safe and cost-effective tools to help determine a person’s risk of lung cancer [16]. There is a clinical need for such a test because more people in the United States die from lung cancer than from any other type of cancer [17]. Early detection can save lives: the National Lung Screening Trial found that screening with low-dose chest CT reduced mortality from lung cancer by 20% [18]. However, the comparatively low positive predictive value (PPV) of chest CT (2.4% to 5.2%) has raised concerns that screening for lung cancer might yield an overwhelming number of false-positive test results [19–21]. An ancillary breath test could potentially improve the sensitivity and specificity of lung cancer screening and reduce the number of false-positive and false-negative test findings.

We designed this study to address two main questions: First, can breath biomarkers of lung cancer identified in one group of subjects predict disease in an independent cohort of subjects with similar demographic features? Second, do breath biomarkers have the potential to add diagnostic value to lung cancer screening with chest CT if the two tests are employed in combination?

**Fig 1:**
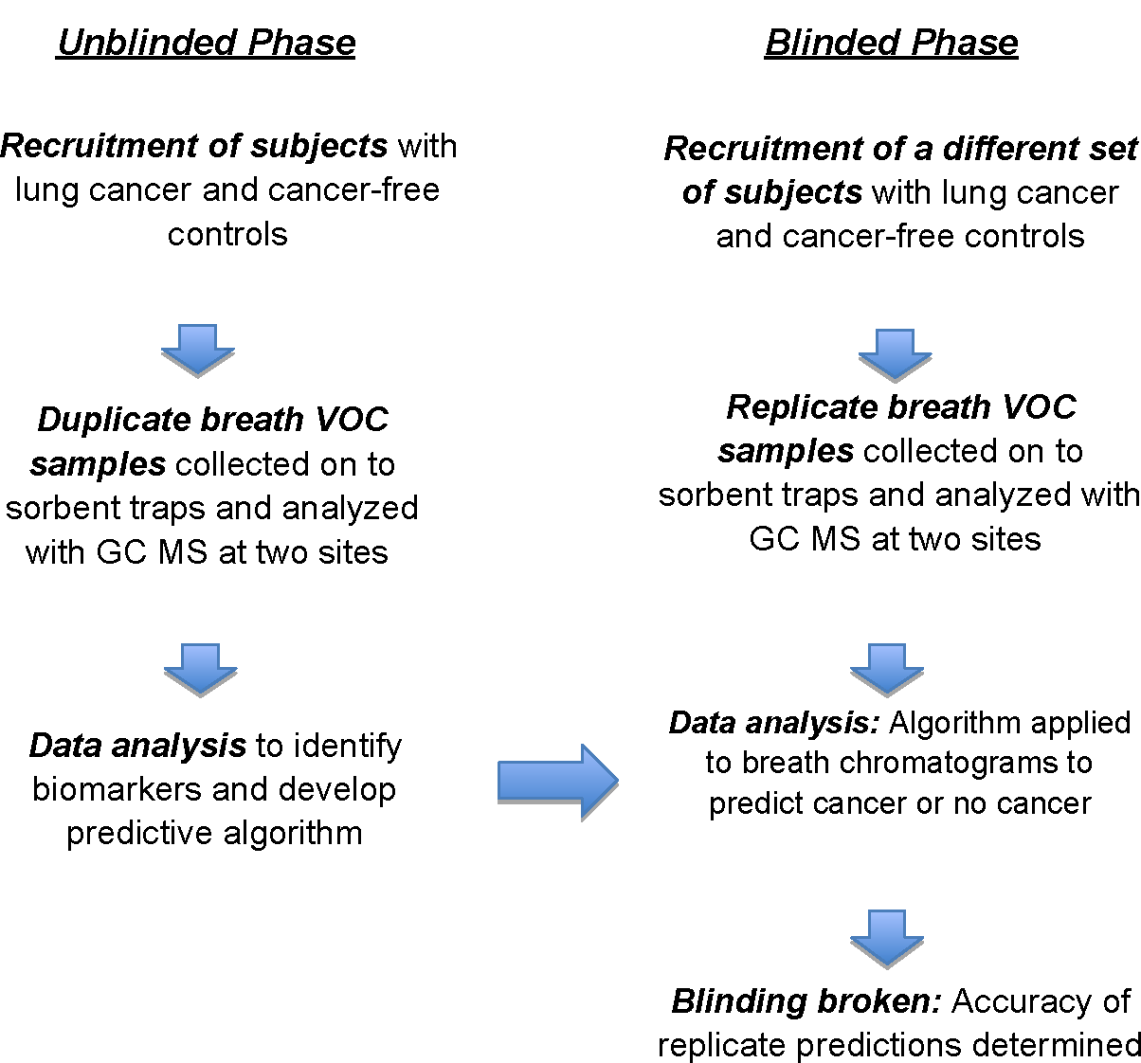
Overview of study design.

We report here a blinded replicated two-phase clinical study of breath biomarkers of lung cancer that was designed to minimize potential sources of error (Fig 1). In the unblinded model-building phase, we analyzed breath samples from subjects with lung cancer and from cancer-free controls with a highly sensitive and selective GC MS assay. A statistical method employing multiple Monte Carlo simulations identified a set of non-random breath biomarkers of lung cancer that were then employed in a multivariate predictive algorithm. In the blinded model-testing phase, we tested the algorithm’s ability to predict lung cancer in a different set of subjects. All breath assays and lung cancer predictions were replicated at two independent analytical laboratories. Additionally, we estimated the potential of breath biomarkers to improve the sensitivity and specificity of lung cancer screening with chest CT when the two tests are employed in combination.

## Methods

### Study design

An overview of the study design is shown in Fig 1. Breath tests were performed in in two phases of human research, and each subject was studied only once. In the unblinded model-building phase of the research, we identified breath biomarkers of lung cancer and combined them in a predictive algorithm. In the blinded model testing phase, we validated the predictive algorithm in a new and independent cohort of subjects. In both phases of the study, four groups were recruited from out-patient volunteers:

#### Group 1

*Asymptomatic high-risk subjects comprising tobacco smokers aged 50 and older undergoing low-dose computed tomographic (chest CT) screening for lung cancer* [22].

#### Group 2

*Symptomatic high-risk subjects without a tissue diagnosis*. These subjects were undergoing medical evaluation for a pulmonary symptom e.g. chronic unexplained cough or hemoptysis that may or may not have been related to an underlying lung cancer. Subjects were transferred into Group 3 if a tissue diagnosis subsequently became available prior to analysis of data.

#### Group 3

*Symptomatic high-risk subjects with a tissue diagnosis of cancer or other pathology*.

#### Group 4

*Apparently healthy subjects*. These subjects were male or female non-smokers with no signs or symptoms of lung carcinoma, aged 18 and older.

## Model-Building Phase -–Unblinded

### Human subjects (Table 1)

**Table 1:** *Human subjects*. Pathological diagnoses employed the 2004 WHO classification.

	Group 1		Group 2		Group 3	Group 4	
	Asymptomatic		Symptomatic		Symptomatic	Healthy	Total
	high-risk smokers		high-risk		high-risk	normals	
	Chest CT		No tissue diagnosis		With tissue diagnosis		
***Model-building phase***							
***Unblinded***							
No.	82		84		100	35	**301**
Age: mean yr (SD)	61.82	(7.24)	64.58	(9.90)	67.72 (10.74)	44.46 (13.72)	
Tobacco smoking:	42.10	(16.89)	36.65	(24.45)	48.49 (28.00)	18.38 (15.83)	
mean pack years (SD)							
Male/female	40/41		30/54		48/52	9/26	
Lung cancer							
positive	1				95		
negative	81				4		
not reported					1		
***Model-testing phase***							
***Blinded***							
No.	70		51		75	19	**215**
Age: mean yr (SD)	62.00	(7.42)	62.78	(12.08)	66.48 (8.9) NS*	49.11 (13.96)	
Tobacco smoking:	43.35	(22.47)	36.34	(31.40)	51.59 (36.6) NS*	11.66 (7.57)	
mean pack years (SD)							
Male/female	31/39		26/25		32/43	9/10	
Lung cancer							
positive	3				73		
negative	65				0		
not reported					2		
***Group 3 tissue diagnosis***			***Model-building phase***		***Model-testing phase***		
			***Unblinded***		***Blinded***		
Adenocarcinoma			53		47		
Adenocarcinoma with			3		0		
bronchioloalveolar carcinoma							
Bronchioloalveolar carcinoma			1		4		
Carcinoid			2		0		
Small cell lung carcinoma			1		0		
Squamous cell lung carcinoma			16		13		
Other or unspecified			1		1		
Other or unspecified			16		8		
non-small cell lung carcinoma							
Mesothelioma			2		0		
***Total***			95		73		

* NS compared to Group 1 (2-tailed t-test assuming equal variances)

Subjects were recruited at five medical centers: Christiana Care Health System, Newark, DE, New York Presbyterian/Columbia University Medical Center, New York, NY, New York University Langone Medical Center, New York, NY, MD Anderson Cancer Center Orlando, Orlando, FL, and Swedish Cancer Institute, Seattle, WA. The Institutional Review Board at all sites approved the study, and all subjects gave their signed and witnessed informed consent to participate.

Subjects with a previously documented history of cancer of any anatomical site were excluded from the study. All data were anonymized with a subject identification number so that no subject could be identified by name. An independent monitor **(**Schiff & Co, West Caldwell, NJ 07006) maintained a clinical database and ensured compliance with regulatory requirements and Good Clinical Practice [23] at all study sites.

#### Collection of breath VOC samples

The method has been described [3,24]. A subject wears a nose clip and breathes normally through a disposable valved mouthpiece and bacterial filter into the BCA for 2.0 min. Alveolar breath VOCs are captured on to a sorbent trap that is immediately sealed in a hermetic container. Since there is low resistance to expiration (~6 cm water), breath samples could be collected without discomfort from elderly patients and those with respiratory disease. In order to minimize the risk of potential site-dependent confounding factors such as environmental contamination of room air, subjects in all four groups donated breath samples in the same room at each clinical site. Samples of breath VOCs and ambient room air VOCs were collected from all subjects in order to control for potential effects of environmental contaminants. Duplicate breath VOC samples were collected from all subjects, for replicate assay at two independent laboratories (Menssana Research, Inc and American Westech, Inc., Harrisburg, PA). Samples were stored at -15°C prior to analysis.

#### Analysis of breath VOC samples

**Fig 2:**
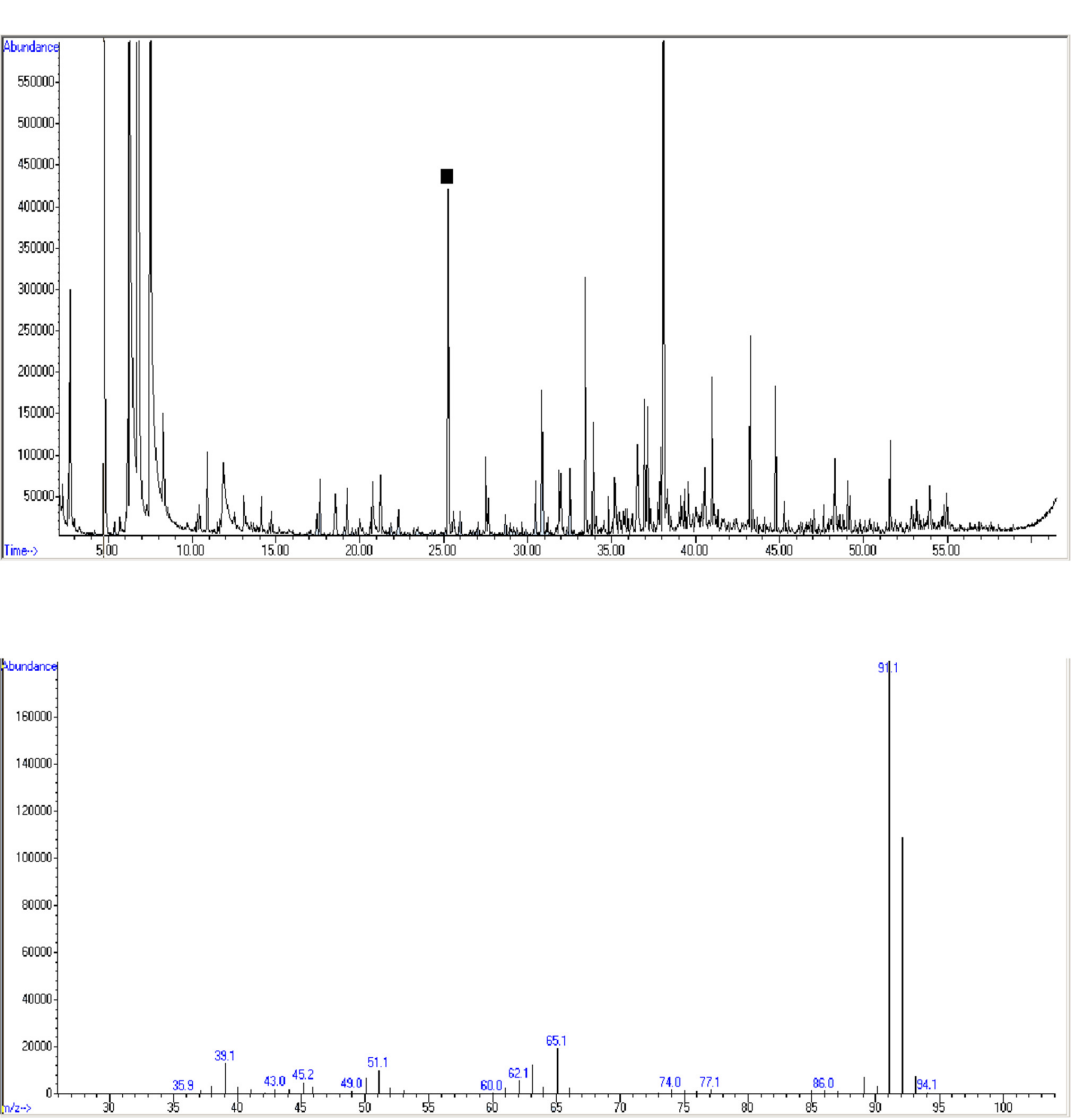
Breath VOC sample analysis. **Total ion chromatogram of breath VOCs (upper panel)** [3, 24]. VOCs are thermally desorbed from the sorbent trap, separated by gas chromatography, and injected into a mass sensitive detector where they are bombarded with energetic electrons in a vacuum and degraded into a set of ionic fragments, each with its own mass/charge (m/z) ratio. This figure displays the total ion current as a function of time, as a series of VOCs enter the detector sequentially. The total ion current from a peak containing toluene is marked, and the mass spectrum of the constituent mass ions is shown in the lower panel. A typical total ion chromatogram derived from a sample of human breath VOCs usually displays ~150 to 200 separate peaks. **Mass spectrum of ions in a chromatograph peak (lower panel).** The mass spectrum of ions derived from toluene (shown in the middle panel) comprises a characteristic pattern of fragments. Matching this pattern to a similar mass spectrum in a computer-based library enables identification of the chemical structure of the source VOC. In complex mixtures like breath, identification is usually tentative because biomarkers may be misidentified if co-eluting VOCs contaminate a mass spectrum, and if the spectral pattern matches inexactly with a library standard. However, individual mass ions from a VOC can be identified with confidence and provide robust biomarkers even when the identity of the parent VOC biomarker is uncertain.

The method has been described [3,24]. Using automated instrumentation, VOCs were thermally desorbed from the sorbent trap, cryogenically concentrated, and assayed by gas chromatography mass spectrometry (GC MS). A known quantity of an internal standard (bromofluorobenzene) was automatically loaded on to all samples in order to normalize the abundance of VOCs and to facilitate alignment of chromatograms. A typical total ion chromatogram of breath VOCs is shown in Fig 2, upper panel. Mass ions detected in a typical chromatograph peak are shown in Fig 2, lower panel.

#### Analysis of data

GC MS data from both laboratories was pooled for analysis and development of a single predictive algorithm.

#### Alignment of individual ion masses in chromatograms

Chromatograms were processed with metabolomic analysis software (XCMS in R [25,26]) in order to generate a table listing retention times with their associated ion masses and intensities. Retention times and ion mass intensities were normalized to the bromofluorobenzene (ion mass 95) internal standard in each chromatogram. The aligned data was then binned into a series of 5 sec retention time segments.

#### Identification of biomarker mass ions

The statistical methods have been previously described [27, 14, 13]. We ranked mass ions as candidate biomarkers of lung cancer by comparing their intensity values in subjects with lung cancer (Group 3 lung cancer confirmed by tissue diagnosis) versus cancer-free controls (Group 1 with negative chest CT). In each 5 sec time segment, the diagnostic accuracy of each mass ion was ranked according to its C-statistic value [(area under curve (AUC) of the receiver operating characteristic (ROC) curve]. We employed multiple Monte Carlo simulations in order to minimize the risk of including random identifiers of disease by selecting the mass ions in each time segment that identified active lung cancer with greater than random accuracy. The average random behavior of mass ions in each time segment was determined by randomly assigning subjects to the ‘‘lung cancer” or the ‘‘cancer-free” group and performing 40 estimates of the C-statistic. For any given value of the C-statistic, it was then possible to identify the ionic biomarkers that exhibited greater diagnostic accuracy with correct assignment than with multiple random assignments.

#### Development of predictive algorithm

Biomarker ions that identified lung cancer with greater than random accuracy were employed to construct a predictive algorithm using multivariate weighted digital analysis (WDA) [28]. WDA is a non-linear method of multivariate analysis that generates a discriminant function to predict membership in a group (disease or no disease) by determining weight (the C-statistic of each predictor variable), a cutoff value, and a sign for each predictor variable employed in the model.

## Model-Testing Phase–Blinded

### Blinding procedures

The independent monitor maintained a database of all clinical and diagnostic data, and this information was not shared with any participant in the research. Laboratories received no clinical information and only the subject identification number accompanied sorbent traps sent for analysis.

#### Human subjects (Table 1)

A new set of human subjects was recruited in the same fashion as described above in the model-building phase. No subject from the unblinded phase was included in the blinded phase of the research.

Collection of breath VOC samples and analysis of breath VOC samples were performed in the same fashion as described above in the model-building phase.

#### Prediction of outcomes

The predictive algorithm developed in the unblinded phase was applied to the mass ions in each of the blinded breath chromatograms in order to generate a discriminant function (DF) value. This procedure was replicated in duplicate breath samples that were analyzed at two laboratories. At the conclusion of the study, the resulting DF values with their associated subject identification numbers were transmitted to the monitor who then broke the blinding and determined the predictive accuracy of the breath test.

## Projected Outcome Modeling

We modeled the expected effects of combining breath biomarkers with chest CT on the sensitivity and specificity of lung cancer screening using a mathematical model to estimate the outcome of combining two different tests for a disease in series and in parallel [29]. The model employed values for sensitivity and specificity of the breath test determined in the blinded model-testing phase, and values reported in the National Lung Screening Trial for lung cancer prevalence (1.1%) and screening chest CT (sensitivity 93.8%, specificity 73.4%) [18].

### Results

#### Human subjects

There were no adverse effects associated with breath testing in either phase of the study.

### Model-Building Phase

**Fig 3:**
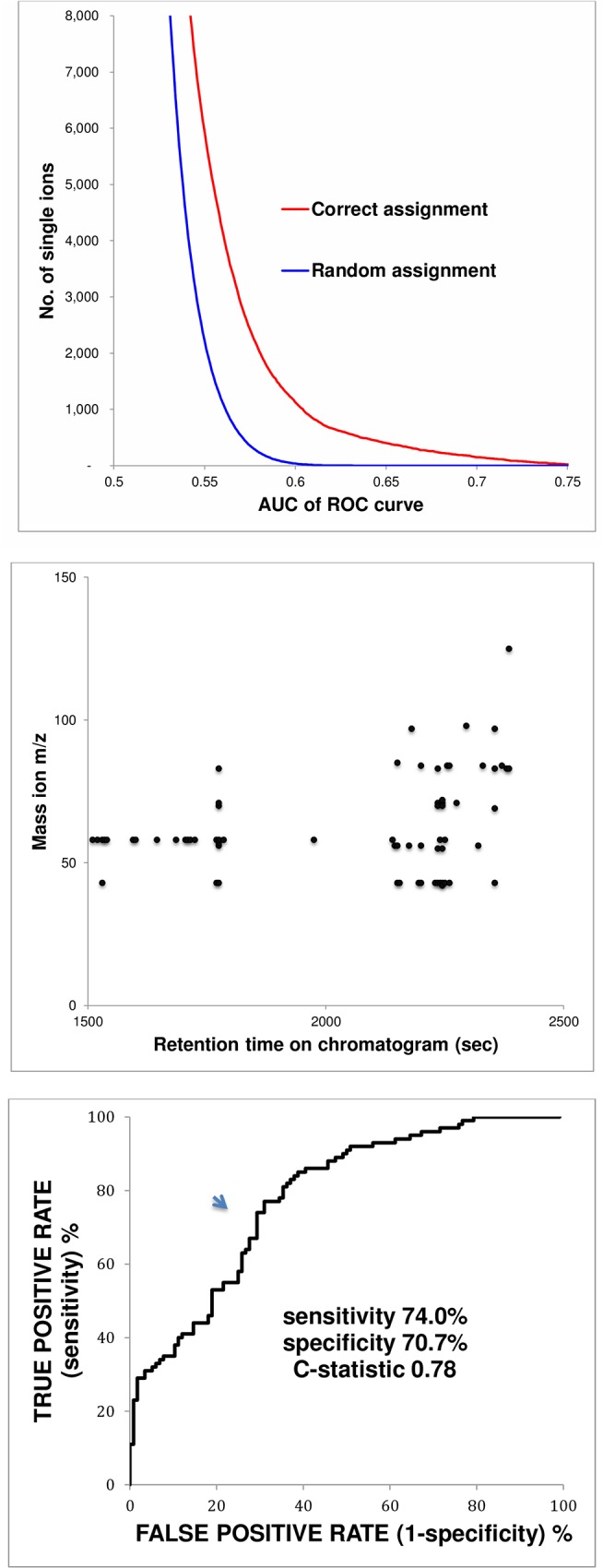
Unblinded development of predictive algorithm. **Monte Carlo statistical analysis of mass ions (top panel)** A list of more than 70,000 candidate mass ion biomarkers of lung cancer was obtained from a series of 5 sec segments in aligned chromatograms. The diagnostic accuracy of each mass ion was quantified by its C-statistic i.e. by the area under curve (AUC) of its associated receiver operating characteristic (ROC) curve (the “Correct assignment” curve). In order to exclude false biomarkers, the ‘‘Random assignment” curve employed multiple Monte Carlo simulations comprising 40 random assignments of diagnosis (“cancer” or “cancer-free”) to determine the random behavior of each candidate mass ion. The cutoff point in the “Correct assignment” curve was taken as the vertical intercept of the point where the number of mass ions in the ‘‘Random assignment” curve declined to zero (at C-statistic = 0.63). At this point, the vertical distance between the two curves indicated that 544 mass ions identified lung cancer with greater than random accuracy, and the separation between the curves exceeded 5 sigma. **Linear clustering of mass ion biomarkers (middle panel).** This figure displays vertical and horizontal linear clustering in a group of mass ion biomarkers of lung cancer with retention times between 1,500 and 2,500 sec. These mass ions were identified by Monte Carlo statistical analysis (upper panel) as having C-statistic values that were greater than random. M/z is the mass divided by the charge number of an ion, and the retention time indicates when a VOC eluted from the GC column and entered the MS detector where it was bombarded with electrons and converted to mass ion fragments. Vertical linear clusters indicate mass ions with similar retention times. These groupings are consistent with one or more breath VOCs entering the MS detector simultaneously, prior to breakdown to mass ions. This observation suggests that a comparatively small number of parent breath VOCs may account for several of the mass ion biomarkers of lung cancer. Horizontal linear clusters with m/z values of 43 and 57 are consistent with breakdown products of alkanes and methylated alkanes. **Receiver operating characteristic (ROC) curve (bottom panel).** The AUC of a ROC curve (or its C-statistic) indicates the overall accuracy of a test, and may vary from 0.5 (a straight line from bottom left to top right of the graph) to 1.0 (a right angle with its apex at the top left of the graph). A C-statistic of 0.5 indicates that the test performance was no better than random e.g. flipping a coin, while a C-statistic of 1.0 indicates a perfect test with 100% sensitivity and specificity. In clinical practice, a C-statistic of 0.78 is generally regarded as clinically useful.

**Monte Carlo statistical analysis of mass ions** (Fig 3, top panel). More than 70,000 mass ions were observed in all of the chromatographic time segments. However, fewer than 1,000 mass ions exhibited useful diagnostic accuracy (C-statistic >0.6) with correct assignment compared to multiple random assignments.

**Linear clustering of mass ion biomarkers of lung cancer** (Fig 3, middle panel). displays vertical and horizontal linear clustering in a group of mass ion biomarkers of lung cancer with retention times between 1,500 and 2,500 sec. *Vertical linear clusters* indicate mass ions with similar retention times, consistent with one or more breath VOCs entering the MS detector simultaneously, prior to breakdown to mass ions, suggesting that a comparatively small number of parent breath VOCs may account for several of the mass ion biomarkers. *Horizontal linear clusters* with similar m/z values (43 and 57) are consistent with breakdown products of alkanes and methylated alkanes.

**ROC curve** (Fig 3, bottom panel). The 500 mass ion biomarkers of lung cancer with the highest C-statistic values were employed in a multivariate WDA algorithm that was applied to all of the chromatograms analyzed at two laboratories. The ROC curve indicated sensitivity 74.0%, specificity 70.7%, and C-statistic 0.78.

### Model-Testing Phase

**Fig 4:**
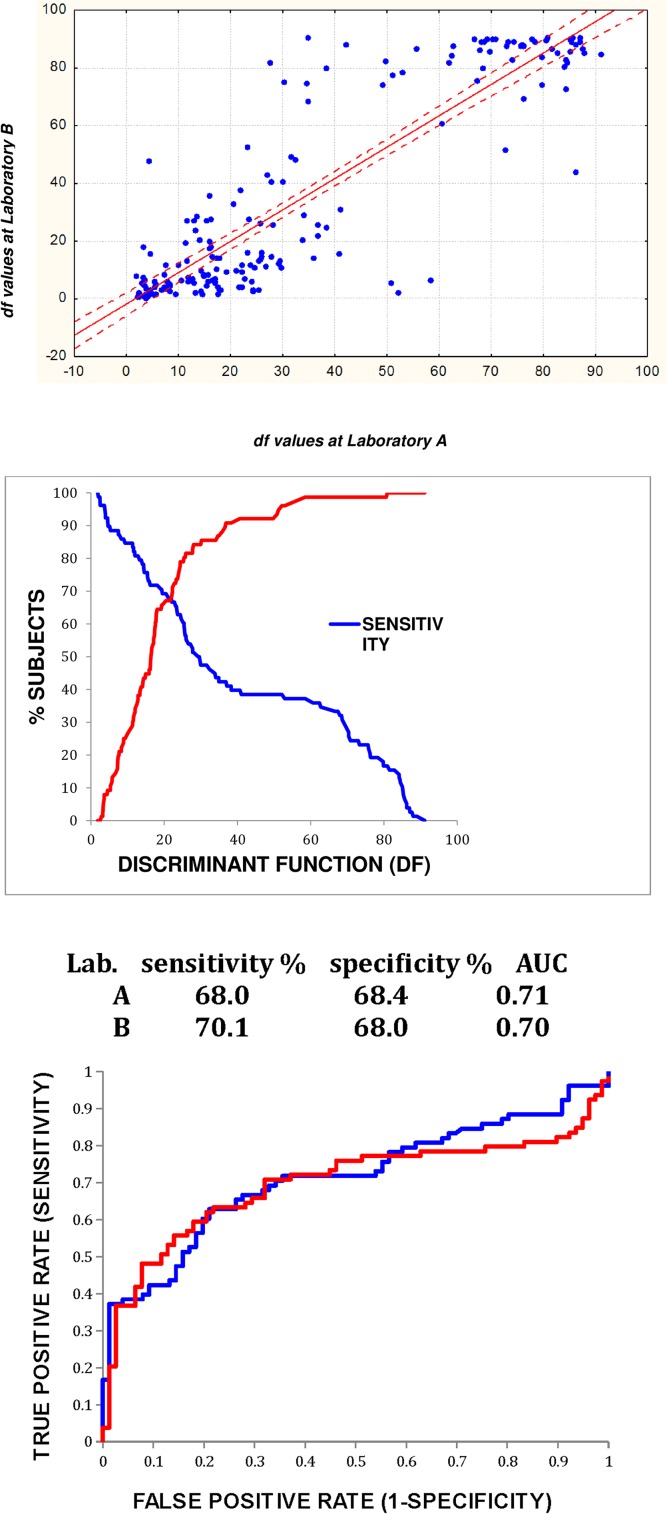
Blinded prediction of lung cancer. **Inter-laboratory concordance of discriminant functions (DF) in replicate samples (top panel).** DF values of chromatograms analyzed at laboratory A were plotted as a function of the DF value of the duplicate sample analyzed at laboratory B. There was a linear relationship between the two sets of DF values (r = 0.88, 95% confidence intervals shown). **Predicted sensitivity and specificity in subjects with biopsy-proven lung cancer and chest CT negative for lung cancer (middle panel).** The DF value derived from the predictive algorithm provides a variable cutoff point for the breath test. Test results greater than a DF value were scored as positive for lung cancer while those less than the DF were scored as negative. When DF = 0, the test has 100% sensitivity because all results are scored as positive for lung cancer, but zero specificity because no results are scored as negative. The sum of sensitivity plus specificity is maximal at the point where the two curves intersect, and was therefore selected as the optimal DF cutoff value for a binary test (i.e. cancer versus no cancer). In this graph (results from Laboratory A), the curves intersected at DF = 22, with sensitivity 68.0% and specificity 68.4%. **ROC curves (lower panel).** The ROC curves of the predicted outcomes of the breath test are shown for samples analyzed at laboratories A and B. The overall accuracy (C-statistic) of the lung cancer predictions was similar at both sites.

The inter-laboratory concordance of predicted discriminant functions in the blinded samples is shown in Fig 4, top panel. There was a linear relationship between DF values derived from samples analyzed at the two laboratories (r = 0.88).

#### Sensitivity and specificity versus discriminant function

Fig 4, middle panel shows predicted outcome in subjects with biopsy-proven lung cancer and chest CT negative for lung cancer. Sensitivity and specificity curves intersected at DF = 22, with sensitivity 68.0% and specificity 68.4%.

**ROC curves** (Fig 4, lower panel). DF values derived from analysis of breath VOC samples at two independent laboratories predicted lung cancer with similar accuracy: Site A sensitivity 68.0% specificity 68.4%, C-statistic 0.73; Site B sensitivity 70.1%, specificity 68.0%, C-statistic 0.70.

#### Effect of age and tobacco smoking

There were no significant differences in age or pack-years of tobacco smoking between the lung cancer group and the cancer-free controls (Table 1).

## Projected Outcomes

**Fig 5:**
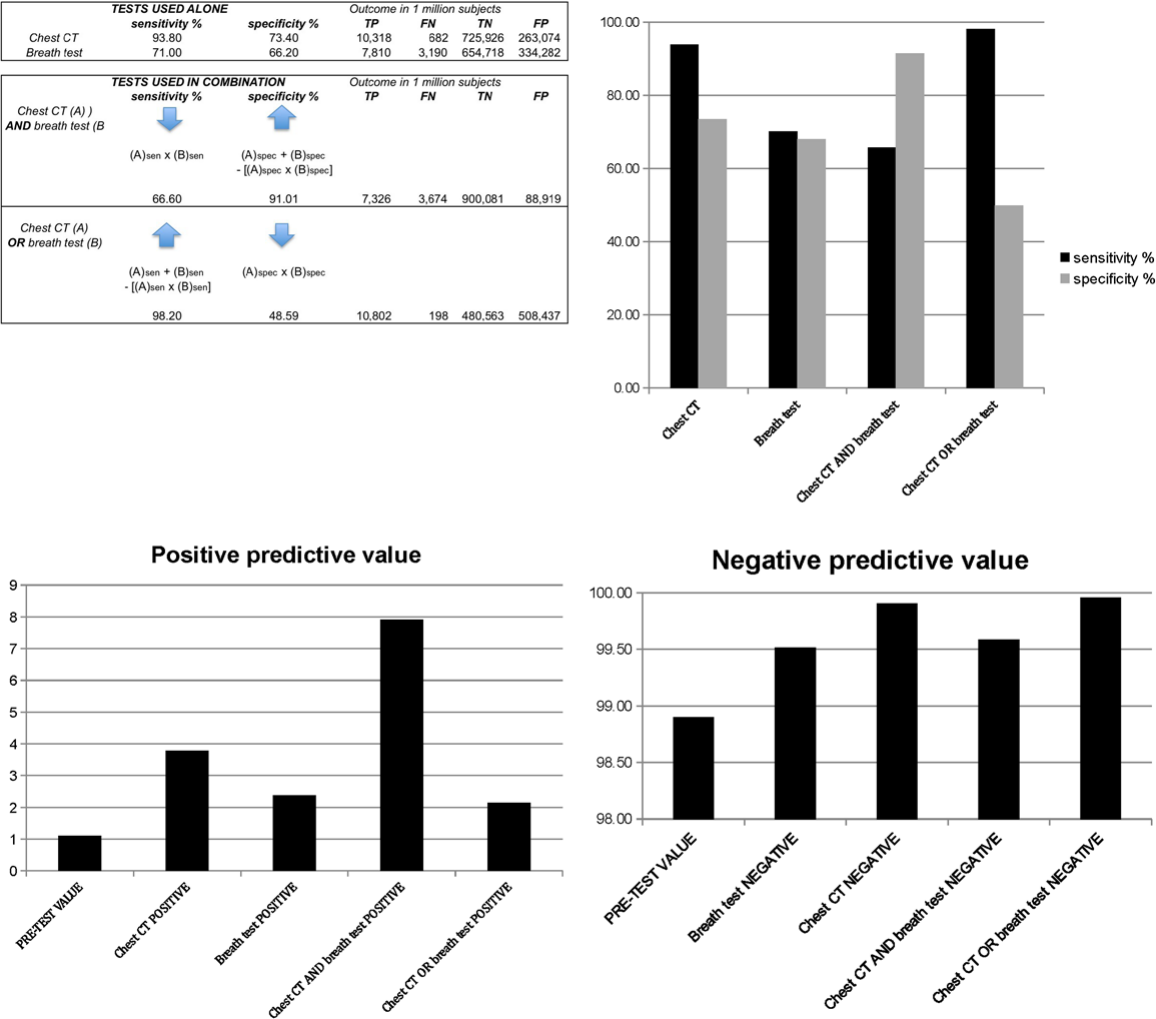
Projected outcome of chest CT combined with breath testing. These predictions employ values reported in the National Lung Screening Trial for lung cancer prevalence (1.1%) and screening chest CT (sensitivity 93.8%, specificity 73.4%) [18]. **Effect of combining two tests (top left panel).** TP = true positives, FN = false negatives, TN = true negatives, FP = false positives. The equations demonstrate the effects on sensitivity and specificity when two tests A and B are combined. If the diagnostic criterion is a positive test result for both test A and test B, then sensitivity decreases and specificity increases, compared to either test employed alone. If the diagnostic criterion is a positive test result for either test A or test B, then sensitivity increases and specificity decreases, compared to either test employed alone. The figure demonstrates the expected outcome of lung cancer screening in one million high-risk people (smokers or former smokers aged 50 yr or older). The main limiting factor in population screening programs is the potentially overwhelming number of false-positive test results. Screening one million people with chest CT alone would result in 263,074 false positive test results, but if chest CT and breath testing are both positive, the increased specificity would reduce this number to 88,919 i.e. by 66.2%. If only one of the tests is positive, then the increased sensitivity would reduce the number of false-negatives from 682 to 198 i.e. by 71.0%. **Effect of parallel and series testing on sensitivity and specificity (top right panel).** This figure displays the expected improvement in sensitivity and specificity of chest CT for lung cancer if it is combined in parallel with a breath testing. If both tests are positive for lung cancer, then specificity increases from 73.4% to 91.49%. If either test is positive, then sensitivity increases from 93.8% to 98.15%. If the two tests are employed in series and the breath test is negative, there may be no need to proceed to chest CT because 98.15% sensitivity is greater than the sensitivity of either test employed alone. **Positive predictive value** (**PPV) of chest CT combined with breath testing (bottom left panel).** This figure displays the expected improvement in PPV of chest CT for lung cancer if combined in parallel with a breath test. Employed alone, the PPV of chest CT is 3.77%. If breath testing is employed in parallel with chest CT and both tests are positive, then the PPV increases to 7.91% i.e. it increases by a factor of 2.1. The improvement is due to the higher specificity of the combined test and the consequent reduction in false positive results. The PPV of a test depends upon the prevalence (prev) of a disease, and is computed as PPV = (sen X prev)/[(sen X prev + (1-spec) X (1-prev)]. The PPV of chest CT for lung cancer is 3.77% [i.e. 0.938 X 011/(0.938 X.011+(1–0.734 X (1–0.011)) = 0.0377]. **Negative predictive value (NPV) of chest CT combined with breath testing (bottom right panel).** If the two tests are employed in series, a negative breath test result rules out lung cancer with NPV 99.6%, which is greater than the NPV of either test employed alone. Despite the increased sensitivity of the combined test, only a modest increment in NPV is possible because the pre-test NPV based on prevalence of lung cancer is 98.9%.

Fig 5 shows the projected outcomes of combining the breath test and chest CT in series and in parallel [29].

If the test results are concordant (i.e. both are negative or both are positive) then the specificity of the combined tests, compared to that of chest CT alone, would increase from 73.4% to 91.01%, and the PPV would increase from 3.77% to 7.91%. If the test results are discordant (i.e. one is negative and the other is positive), then the sensitivity of the combined tests, compared to that of chest CT alone, would increase from 93.8% to 98.2%, and the NPV would increase from 99.52% to 99.6%. In the projected outcome of screening one million people, the increased sensitivity and specificity would be expected to reduce the false-positive rate of chest CT by 66.2% and the false-negative rate by 71.0%.

## Discussion

Ionic biomarkers in breath predicted the presence or absence of lung cancer in a blinded validation study. A multivariate algorithm predicted the diagnosis from replicate breath samples independently analyzed at two laboratories, and the sensitivity, specificity, and overall accuracy of the test were similar at both sites. The outcome of the test was not significantly affected by age or pack-years of tobacco smoking.

This is the first report of validation of breath biomarkers of lung cancer in a blinded replicated study. The earliest evidence for breath VOC biomarkers of lung cancer was reported by Gordon et al in 1985 [4], followed by Preti et al in 1988 [6], then by several other reports from our group and from other investigators. These studies generally followed a similar approach to biomarker discovery by analyzing breath VOCs in subjects with histologically-proven lung cancer and in cancer-free controls, then comparing the two groups for statistically significant differences. A number of these studies claimed that breath VOCs identified lung cancer with sensitivity and specificity values approximately similar to those observed in this report, with ROC curve AUC values of 0.7 to 0.9. However, all were susceptible to false-positive identifications of biomarkers of lung cancer, and none of the candidate biomarkers were subsequently validated in a separate set of patients. This study minimized these sources of error, first by a rigorous statistical screening to identify non-random biomarkers, and second, by validating the biomarkers of lung cancer in a blinded replicated study in a new set of patients.

The breath test for biomarker ions could potentially improve the sensitivity and the specificity of chest CT as well as its positive and negative predictive values, if the two tests are employed in combination [29]. If the diagnostic criterion is a positive test result for both the breath test and for chest CT, then sensitivity decreases and specificity increases, compared to either test employed alone. If the diagnostic criterion is a positive test result for either the breath test or chest CT, then sensitivity increases and specificity decreases, compared to either test employed alone. In clinical practice, breath testing and chest CT could provide a synergistic combination with greater diagnostic accuracy than either test employed alone.

A program to screen one million asymptomatic high risk-subjects for lung cancer with chest CT alone would be expected to generate 263,074 false-positive test results. However, if chest CT and a breath test were combined in parallel (i.e. breath test and chest CT positive), the number of false-positive results would be expected to fall to 88,919, a reduction of 66.2%. Similarly, if only one of the tests is positive, then the number of false-negatives would be expected to fall from 682 to 198 i.e. by 71.0%. As a result, combined use of the two tests could potentially facilitate large-scale screening for lung cancer by reducing the number of needless additional tests that are currently performed. This could reduce the economic costs and the potential harms of false-positive and false-negative test outcomes that are currently associated with chest CT screening for lung cancer [20,21].

It was not possible to test the predicted outcomes of combined testing because this study was not designed as a prospective evaluation of chest CT, and the number of false-negative and false-positive results of chest CT was insufficient for statistical analysis.

GC MS analysis of breath has historically focused on identifying the chemical structure of exhaled VOCs by reference to a large library of mass spectra, but this has proved challenging for two main reasons: First, it is technically difficult to resolve pure VOC peaks without simultaneous co-elution of different compounds. Second, even when there is no apparent co-elution of a VOC, its chemical structure cannot always be identified with confidence because of the uncertainty inherent in matching a complex mass spectrum of ionic fragments to a corresponding mass spectrum in a large library. The pitfalls in compound identification that arise from searching mass spectral reference libraries include the risks of false-negative and false-positive results [30]. As a result, GC MS usually provides only tentative structural identification of VOCs, which has limited the reproducibility and diagnostic value of previously reported breath VOC biomarkers.

However, even if the chemical structure of a parent VOC is not known with certainty, its ionic daughter products appear to provide stable and robust biomarkers because they are not affected by co-elution, nor do they require reference to a mass spectral library. The observation of vertical linear clusters of ionic biomarkers (Fig 3) was consistent with daughter products of a comparatively small number of parent breath VOCs with different chromatographic retention times. However, tentative identification of their parent VOCs will require further study because a number of significant VOC biomarkers appeared to co-elute with one another, with consequent mixing of their ionic signatures. Other studies have also reported ionic biomarkers of lung cancer, employing instruments that ionize breath VOCs without preliminary GC separation [31,32].

The metabolic origin of breath biomarkers of lung cancer is unknown. Various mechanisms have been proposed, including modulation of oxidative stress, production of reactive oxygen species and alkane production, and modulation of cytochrome p450 hepatic enzymes [33]. Several open questions continue to attract research in this field e.g. some VOC biomarkers associated with lung cancer appear to be produced by malignant cells [34], but other possible origins of the VOCs are also under investigation, including the cancer microenvironment and extra-pulmonary sources.

Candidate biomarkers in breath may be affected by the presence of concomitant conditions that are also known to modify the composition of breath VOCs e.g. diseases of liver [35], kidney [36], asthma [37], or COPD [38]. It was not feasible to stratify the statistical analysis of data for every potential confounding variable since the large number of subgroups would have reduced the statistical power of the study. For this reason, we adopted a strategy that is commonly employed in randomized clinical trials of therapeutic interventions [39,40]: since the same recruitment criteria were employed in both the blinded and unblinded studies and there were no significant differences between age, sex, and tobacco smoking in both groups, we therefore assumed that the a priori probability of any concomitant disease would be similar in both groups, so their presence would not skew the outcome of the study.

Different methodologies have been reported for the collection of concentrated breath samples in order to control for effects of anatomy [41,42] (i.e. dilution of alveolar breath with dead space air from the upper airways) and of physiology e.g. flow rate volume of exhalation, exhalation with or without breath holding, exhalation in single or multiple breathing and volume of air inhaled before breath gas exhalation [43]. The breath collection apparatus employed in this study controlled for anatomical effects by sampling alveolar breath that had been separated from dead space air in a tubular breath reservoir [3,44]. We controlled for potential physiological confounders by requiring subjects to sit quietly for at least 15 min prior to donating a sample, and respiring normally into the device while seated comfortably. Breath samples were collected without distress from subjects who were elderly or who suffered from respiratory disease because low resistance in the breath reservoir did not impede normal tidal respiration.

The optimal methodology for sampling breath for biomarkers of lung cancer remains unresolved because there is evidence for both local and systemic origins of these VOCs. Studies of VOCs derived from lung cancer cells [45] and of VOCs derived from one-lung sources [46] provide evidence that VOCs may be produced by tumor tissue both in vivo and in vitro. However, accelerated catabolism of alkane products of oxidative stress in lung cancer is consistent with an extrapulmonary process, such as the induction of cytochrome p450 mixed oxidase ennzymes [47].

We conclude that breath VOC ionic biomarkers predicted lung cancer in a blinded replicated study. Breath testing in combination with chest CT could potentially improve the accuracy of lung cancer screening.

## Supporting Information

S1 File Fig 3 Middle. Data underlying the middle panel of Fig 3.
(XLSX)

S2 File Fig 3 Lower. Data underlying the lower panel of Fig 3.
(XLSX)

S3 File Fig 4 Top. Data underlying the top panel of Fig 4.
(XLSX)

S4 File Fig 4 Middle. Data underlying the middle panel of Fig 4.
(XLSX)

S5 File Fig 4 Lower. Data underlying the Lower panel of Fig 4.
(XLSX)

S6 File Fig 5 Top R. Data underlying the top-right panel of Fig 5.
(XLSX)

S7 File Fig 5 Bottom R. Data underlying the bottom-right panel of Fig 5.
(XLSX)

S8 File Fig 5 Bottom L. Data underlying the bottom-left panel of Fig 5.
(XLSX)
